# Supplementary material for: A Comprehensive Assessment of Ultraviolet-Radiation-Induced Mutations in Flammulina filiformis Using Whole-Genome Resequencing
Source: J Fungi (Basel). 2024 Mar 20;10(3):228. doi: 10.3390/jof10030228 (PMC10971301; doi:10.3390/jof10030228)
Supplement: Supplementary file 1 [file jof-10-00228-s001.zip › Supplementary Material S8/KEGG annotation/out/64381550635650.os/KO/out_map/map04122.html]

KEGG PATHWAY: Sulfur relay system - Reference pathway


|  |  |
| --- | --- |
| **Sulfur relay system - Reference pathway** |  |

[
Pathway menu
| Organism menu
| Pathway entry
| Show description
| User data mapping
]

Ubiquitin and ubiquitin-like proteins (Ubls) are signalling messengers that control many cellular functions, such as cell proliferation, apoptosis, and DNA repair. It is suggested that Ub-protein modification evolved from prokaryotic sulfurtransfer systems. Molybdenum cofactor (Moco) and thiamin are sulfur-containing cofactors whose biosynthesis includes a key sulfur transfer step that uses unique sulfur carrier proteins, MoaD and ThiS. Ubiquitin, MoaD, and ThiS are all structurally related proteins whose C-termini are activated through adenylation by homologous E1-like enzymes. s2T biosynthesis may share similar chemistry with Moco and thiamin synthesis. In Saccharomyces cerevisiae, Urm1 and Uba4 function as part of a ubl protein conjugation system, though they have sequence homology to bacterial sulfur-transfer enzymes and the ability to function in sulfur transfer.

|  |  |
| --- | --- |
| Reference pathway | 100% |
